# Cardiac Magnetic Resonance Imaging Used to Determine a Rare Etiology of a Layered Left Ventricular Apical Thrombus

**DOI:** 10.7759/cureus.57257

**Published:** 2024-03-30

**Authors:** Valentina Turbay-Caballero, Rachel Morris, Sheraz Hussain, Suyashi Singh, Manuel Paredes-Flores, Shermeen Memon, Amir Naqvi

**Affiliations:** 1 Internal Medicine, Advocate Christ Medical Center, Oak Lawn, USA; 2 Cardiology, Advocate Christ Medical Center, Oak Lawn, USA

**Keywords:** heart failure with reduced ejection fraction., cardiac magnetic resonance imaging, left ventricular thrombus, idiopathic hypereosinophilic syndrome, eosinophilic myocarditis

## Abstract

Eosinophilic myocarditis (EM) is a rare disease, often associated with hypereosinophilic syndrome (HES). Historically, the diagnostic gold standard was endomyocardial biopsy (EMB). We present a unique case of a 58-year-old female who presents after a syncopal episode and was found to have a layered left ventricular (LV) thrombus. Using laboratory studies and cardiac magnetic resonance imaging (MRI), we were able to delineate the etiology, avoiding any invasive testing.

## Introduction

Eosinophilic myocarditis (EM) is a disease characterized by eosinophil infiltration of the myocardium and is often associated with hypereosinophilic syndrome (HES) [1-7]. EM can manifest anywhere from mild pericarditis to restrictive cardiomyopathy given the compromise of either pericardium, myocardium, or endocardium [1,2]. It can promote an inflammatory and prothrombotic state that can subsequently cause fibrosis [2]. Historically, the diagnostic gold standard has been endomyocardial biopsy (EMB), but its use has significantly decreased due to the availability of newer and noninvasive imaging studies that have largely replaced invasive procedures in general [8]. Cardiac MRI is able to characterize endomyocardial fibrosis and intracardiac thrombi, therefore its utility in diagnosing EM [8]. EM treatment will vary based on the etiology and complications of the disease [1-3].

We describe a case of a patient with severely reduced ejection fraction associated with a layered left ventricular (LV) thrombus that with appropriate use of laboratory/imaging studies led to the diagnosis of the underlying pathophysiology.

## Case presentation

A 58-year-old Hispanic female with no known past medical history presented with syncope. She reported a one-month history of abdominal pain, diarrhea, dyspnea on exertion, and confusion. No use of medications, herbal/dietary supplements, alcohol, recreational drugs, or smoking was reported. She did not report any pertinent family history. The patient was tachycardic to 105 beats per minute and reminder of vital signs and physical exam were normal.

The patient’s relevant laboratory test results are depicted in Table 1.

**Table 1:** Initial laboratory results.

Laboratory test	Patient result	Normal range
High-sensitivity troponin I	505	<52 ng/L
Hemoglobin	16.3	12 – 15.5 g/dL
Hematocrit	52.6	36 – 46.5%
Mean corpuscular volume	75.5	78 – 100 fl
White blood cell count	39.1	4.2 – 11 K/mcL
Absolute eosinophil count	30.1	0 – 0.5 K/mcL
Platelets	400	140 – 450 K/mcL
Erythrocyte sedimentation rate	25	0 – 20 mm/hr
C-reactive protein	4.5	<1 mg/dL
Creatinine	0.82	0.51 – 0.95 mg/dL
Glomerular filtration rate	83	>60 mL/min/1.73m^2^
NT-proBNP	6,730	<125 pg/mL
Thyroid-stimulating hormone	2.178	0.350 – 5.000 mcUnits/mL

**Figure 1:**
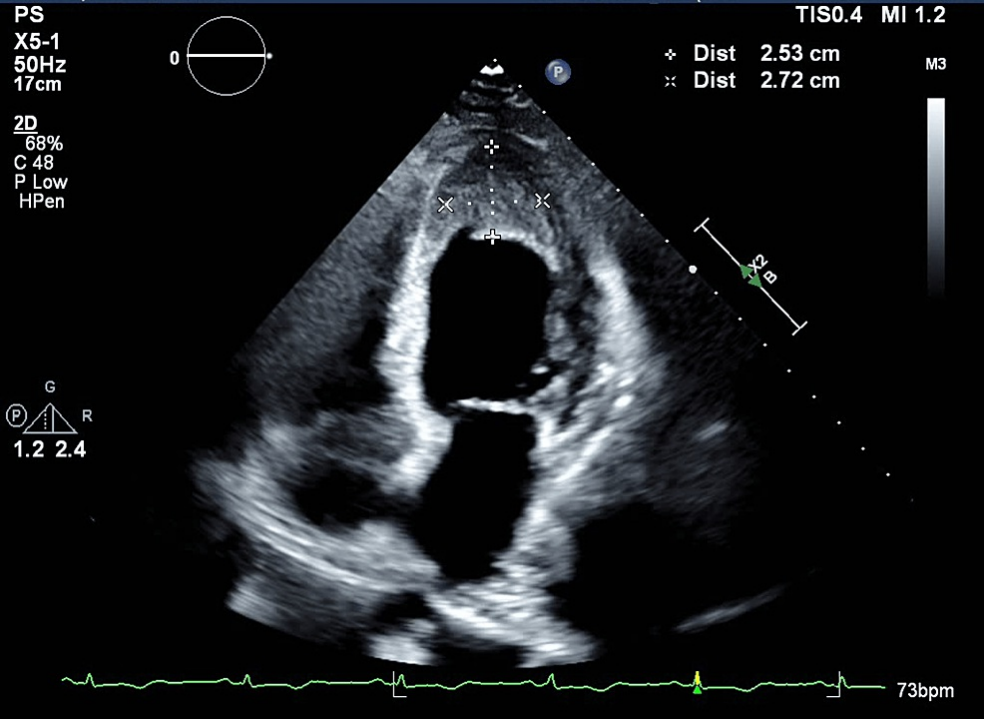
Transthoracic echocardiogram (apical 4-chamber window). Severely hypokinetic apex with a left ventricular ejection fraction of 34% and an associated large, dense, and layered apical thrombus (highlighted in the figure).

An electrocardiogram (ECG) showed normal sinus rhythm with T-wave inversions in anterolateral leads. Chest x-ray was unremarkable. Chest computed tomography angiogram (CTA) excluded pulmonary embolism. Transthoracic echocardiography (TTE) showed a severely hypokinetic apex with an associated large, dense, and layered apical thrombus and LV ejection fraction of 34% (Figure 1). 

**Figure 2:**
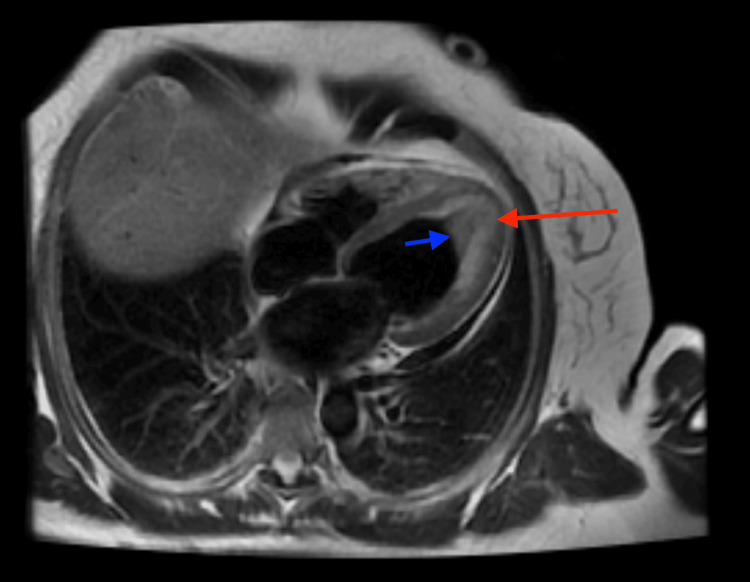
Cardiac MRI (T2 imaging, 4-chamber view). T2 weighted image demonstrates areas of hyperintensity in the left ventricular apex suggestive of underlying edema and inflammation (red arrow) resulting in complete obliteration of the apex. The overlying area of hypointensity was suspicious for an organized apical thrombus (blue arrow). T1 weighted image demonstrated significant late gadolinium enhancement of the left ventricular apical cavity consistent with a background of myocardial fibrosis and scarring (not depicted).

As there was evidence of an intraventricular thrombus, intravenous continuous heparin infusion was initiated. CTA of the coronary arteries showed mild non-obstructive disease of the mid-left anterior descending artery and a hypokinetic LV apical segment with a densely layered thrombus. All the imaging findings along with peripheral eosinophilia, prompted further evaluation via cardiac MRI (Figure 2). MRI noted endomyocardial inflammation and fibrosis resulting in complete obliteration of the LV apex with an overlying thrombus, consistent with eosinophilic myocarditis. High-dose steroids were started after ruling out systemic parasitic infections.

No evidence of active infection was noted on blood, stool, and urine cultures. Testing for HIV, *Strongyloides*, respiratory viral panel, *Clostridium difficile*, *Shigella*, and *Campylobacter* was negative. An autoimmune panel including antinuclear antibodies, myeloperoxidase antibodies, and proteinase-3 antibodies was negative. Immunoglobulin levels were normal. Esophagogastroduodenoscopy and colonoscopy with multi-segment tissue biopsies excluded gastrointestinal involvement. MRI brain showed multiple small punctate foci of acute/subacute infarction in bilateral cerebral hemispheres primarily involving the frontal and parietal lobes, likely cardioembolic in etiology. The coagulation panel included a normal international normalized ratio (INR) of 1.3, slightly prolonged prothrombin time at 13.7 seconds (normal range 9.7-11.8 seconds), and normal partial thromboplastin time at 30 seconds (normal range 22-30 seconds).

Flow cytometry analysis of the peripheral blood showed a population of mature myeloid cells without blasts, gating on the lymphocytes showed predominantly T-cells with a T- to B-cell ratio of 3.5:1 and no monoclonal B-cells or T-cell antigenic abnormalities. A bone marrow core biopsy from the right iliac crest showed slight hypercellularity (60% cellular) with marked eosinophilic hyperplasia. Next-generation sequencing was positive for myeloid malignancy including DNMT3A p.(R882H), JAK2 p.(L583_A586delinsQ), and CEBPA p.(G223S). Multilineage hematopoiesis, M:E ratio-2.8:1 was noted. The fluorescence in situ hybridization (FISH) test was negative using a panel for hypereosinophilia containing probes for 4q12 (SCFD2, LNX, and PDGFRA) rearrangement, 5q32 (PDGFRB) rearrangement, 8p11.2 (FGFR1) rearrangement, and 9q34 and 22q11.2 (BCR/ABL1) rearrangement (direct preparation). These overall findings were consistent with chronic eosinophilic leukemia. 

Her presentation was deemed consistent with eosinophilic myocarditis associated with idiopathic hyper-eosinophilic syndrome (chronic eosinophilic leukemia), complicated by an LV thrombus, heart failure with reduced ejection fraction (HFrEF) and cardioembolic stroke. She continued prednisone 60 mg daily for a total of ten days, in addition to indefinite anticoagulation with warfarin. She was also started on guideline-directed medical therapy (GDMT), with a plan for outpatient follow-up.

The patient had an uneventful recovery after completing a 10-day course of prednisone, she was continued on warfarin, aspirin, and metoprolol tartrate 25 mg twice daily. Repeat TTE revealed persistent large apical thrombus 2.5 cm (L) x 2.7 cm (W), improved ejection fraction of 45%, and a trivial pericardial effusion. A TTE performed one month later revealed similar findings. Repeat follow-up laboratories are shown in Table 2.

**Table 2:** Follow-up laboratory results

Laboratory test	Patient result after two weeks	Patient result after one year	Normal range
Hemoglobin	17	18.2	12 – 15.5 g/dL
Hematocrit	53.2	58.7	36 – 46.5%
Mean corpuscular volume	77.2	73.7	78 – 100 fl
White blood cell count	61.9	68.2	4.2 – 11 K/mcL
Absolute eosinophil count	48.3	40.9	0 – 0.5 K/mcL
Platelets	307	234	140 – 450 K/mcL

For over a year, her clinical course has remained relatively unchanged. She has had regular outpatient followup with cardiology, advanced heart failure, and hematology/oncology. She completed her cancer screening with a mammogram and Pap smear that was normal. Despite long-term steroid use with prednisone 1 mg/kg, her latest complete blood count (CBC) results (exhibited in Table 2), demonstrate persistently elevated eosinophilic count and hematocrit. Additionally, she was started on weekly phlebotomy for a goal hematocrit of 45-50%. Given labile INR levels, she was transitioned from warfarin to apixaban 5 mg twice daily. GDMT was optimized with the addition of sacubitril/valsartan 24/26 mg twice daily. She has had no recent imaging. The plan is to repeat a cardiac MRI when the eosinophilic count and hematocrit improve.

## Discussion

HES is a group of disorders characterized by an overproduction of eosinophils, in which eosinophilic infiltration leads to cytokine-mediated damage of multiple organs [1-5]. Eosinophils normally combat parasites and participate in hypersensitivity and allergic responses [6]. The tissues most often affected by HES include the skin (i.e., recurrent urticaria and angioedema), lungs (i.e., parenchymal infiltrates and pleural effusion), and gastrointestinal tract (i.e., gastritis, enteritis, and colitis) [6]. Eosinophilia-associated cardiac disease was first described by Loeffler [7,9,10] and is present in approximately 20% of patients with HES [6,11-12].

The cardiotoxic effects of eosinophilia are mainly caused by the secreted granulated proteins [13]. It can start as an inflammatory reaction at the level of the pericardium, myocardium, or endocardium lasting weeks, followed by a prothrombotic state where layered thrombi form due to activation of tissue factor by the eosinophils. After months, the damage can progress to myocardial fibrosis causing wall stiffness that can lead to restrictive cardiomyopathy and valvular abnormalities within a couple of years [2,13-15]. Our patient presented with syncope and mildly elevated troponin. She was found to have myocarditis, an LV thrombus, and severe systolic dysfunction. Given limited access and the risk of complications from a myocardial biopsy, cardiac MRI has become more popular of late, as a noninvasive method to visualize the extent of tissue involvement, easily demonstrating bright diffuse enhancement along with intracavitary thrombi as seen in our patient [8,13,16-19]. Cardiac MRI can also be used for follow-up and assessment of response to therapy [20,21]. 

Management should aim to treat the identified problems. For example, our patient required GDMT for heart failure, steroids to control the inflammatory cascade, and anticoagulation to prevent new cardioembolic infarcts [8]. Patients with HES should undergo workup for FIPIL1-PDGFRA mutation. If patients test positive for the FIPIL1-PDGFRA mutation (a treatment-responsive mutation in primary eosinophilia, associated with underlying systemic mastocytosis), treatment with tyrosine kinase inhibitors could be offered [22,23,24]. Unfortunately, for those who do not have this mutation, there is no significant response to tyrosine kinase inhibitors. In these patients, initial therapy is corticosteroids. In case of a lack of response, combination therapy with hydroxyurea is recommended [25]. Other therapies that have been used include hydroxycarbamide, interferon alpha, and cytotoxic chemotherapy [12], however, there is insufficient evidence to support their use.

Other underlying causes of eosinophilic myocarditis should also be identified prior to treatment initiation. Classification of HES is based on three separate categories according to etiology: (i) idiopathic, in which no known cause is identified; (ii) secondary to an underlying cause, including infection (parasitic, viral, helminth), allergies, metastatic disease, autoimmune, endocrinopathies; (iii) and clonal disorders [26-28]. There are several drugs that can lead to a hypersensitivity response resulting in EM including, but not limited to ampicillin, indomethacin, acetazolamide, and dobutamine [26,29,30]. Classic infective causes of myocarditis are viral, most notably Coxsackie B virus [29]. However, especially for eosinophilia, a parasitic cause should be excluded. In approximately 10-15% of *Toxocara canis/cati* infections (a common roundworm found in dogs and cats), patients will develop myocarditis. In this case, albendazole would be the first-line therapy, likely in combination with corticosteroids [27]. A rare complication of bronchial asthma is EM, due to eosinophilic accumulation around the heart and lungs, especially in asthmatics who have transitioned from steroid therapy to anti-leukotriene drugs [31,32]. 

After extensive workup in our patient, a specific trigger for HES was not identified, and she was therefore classified as idiopathic HES with cardiac involvement.

## Conclusions

It is not uncommon to see left ventricular thrombi in patients with heart failure with severely reduced ejection fraction. However, in cases where heart failure or left thrombus formation is present in a patient with eosinophilia, there should be a high suspicion of eosinophilic myocarditis (EM), and a cardiac MRI should be considered as a diagnostic test. EM can be associated with hypereosinophilic syndrome, atopy, malignancy, parasitic or viral infections.
